# Cement leakage into the paravertebral venous system and pulmonary cement embolism following percutaneous vertebroplasty: a case report

**DOI:** 10.3389/fsurg.2025.1643103

**Published:** 2025-10-21

**Authors:** Chengrong Lai, Qingcun Meng, Honglei Zhang, Yanbin Liu, Jinlong Liu

**Affiliations:** 1 The Second School of Clinical Medicine, Binzhou Medical University, Yantai, China; 2 Department of Orthopedics, Liaocheng People’s Hospital, Liaocheng, China; 3 Department of Orthopedics, Beijingjishuitan Hospital Liaocheng Hospital, Liaocheng, China

**Keywords:** percutaneous vertebroplasty, pulmonary cement embolism, bone cement leakage, paravertebral venous plexus, case report

## Abstract

Pulmonary cement embolism is a potentially serious complication following percutaneous vertebroplasty. The risk factors for pulmonary cement embolism have not been clearly identifed. Cement leakage into the azygos vein or in the inferior vena cava has been identifed as a risk factor for pulmonary cement embolism. In addition cement leakage into the paravertebral venous system also seems to be related to pulmonary cement embolism. Here we report a case with detailed preoperative data, intraoperative findings, and imaging evidence to demonstrate the relationship between cement leakage into the paravertebral venous system and pulmonary cement embolism.

**Case:**

A 69-year-old woman was diagnosed with osteoporotic vertebral compression fractures and treated with percutaneous vertebroplasty. Intraoperatively, anterior cement leakage into the paravertebral venous system was detected on lateral fluoroscopy but it did not cause insufficient attention. The patient demonstrated significant back pain relief postoperatively, and had no symptoms such as dyspnoea, coughing, haemoptysis, dizziness or palpitation. However, the postoperative thoracolumbar x-ray presented that multiple tubular and branching cement emboli were scattered throughout the lungs. The patient presented asymptomatically in the follow-up period. No cardiorespiratory dysfunction was observed until the end of the one-year clinical follow-up, and the patient was satisfied with pain relief.

**Conclusion:**

Cement leakage into the paravertebral venous system is associated with pulmonary cement embolism during percutaneous vertebroplasty. Continued bone cement injection after bone cement leakage into the paravertebral venous system is dangerous and, may lead to further migration and, ultimately, pulmonary cement embolism. Once bone cement leakage into the parav ertebral venous system is detected, percutaneous vertebroplasty should be terminated.

## Introduction

Percutaneous vertebroplasty has been widely used for painful osteoporotic vertebral compression fractures since it was first introduced in 1984 (1). Most complications are related to the leakage of bone cement (polymethylmethacrylate), of which pulmonary cement embolism is deemed serious. The observed incidence of pulmonary cement embolism varies from 2.1% to 26% (2–4). Although most pulmonary cement embolisms are asymptomatic (5–10), serious and fatal sequelae have been reported (11–14). Risk factors identified as predictors of pulmonary cement embolism are not clear, with the exception of the presence of cement in the azygos vein or in the inferior vena cava. Cement leakage into the paravertebral venous system also seems to be related to pulmonary cement embolism, but there is not sufficient supporting clinical evidence. Here, we present a new case that confirmed the relationship between cement leakage into the paravertebral venous system and pulmonary cement embolism. In addition we elaborate that continued bone cement injection after bone cement leakage into the paravertebral venous system can lead to misoperation.

## Case presentation

**Figure 1:**
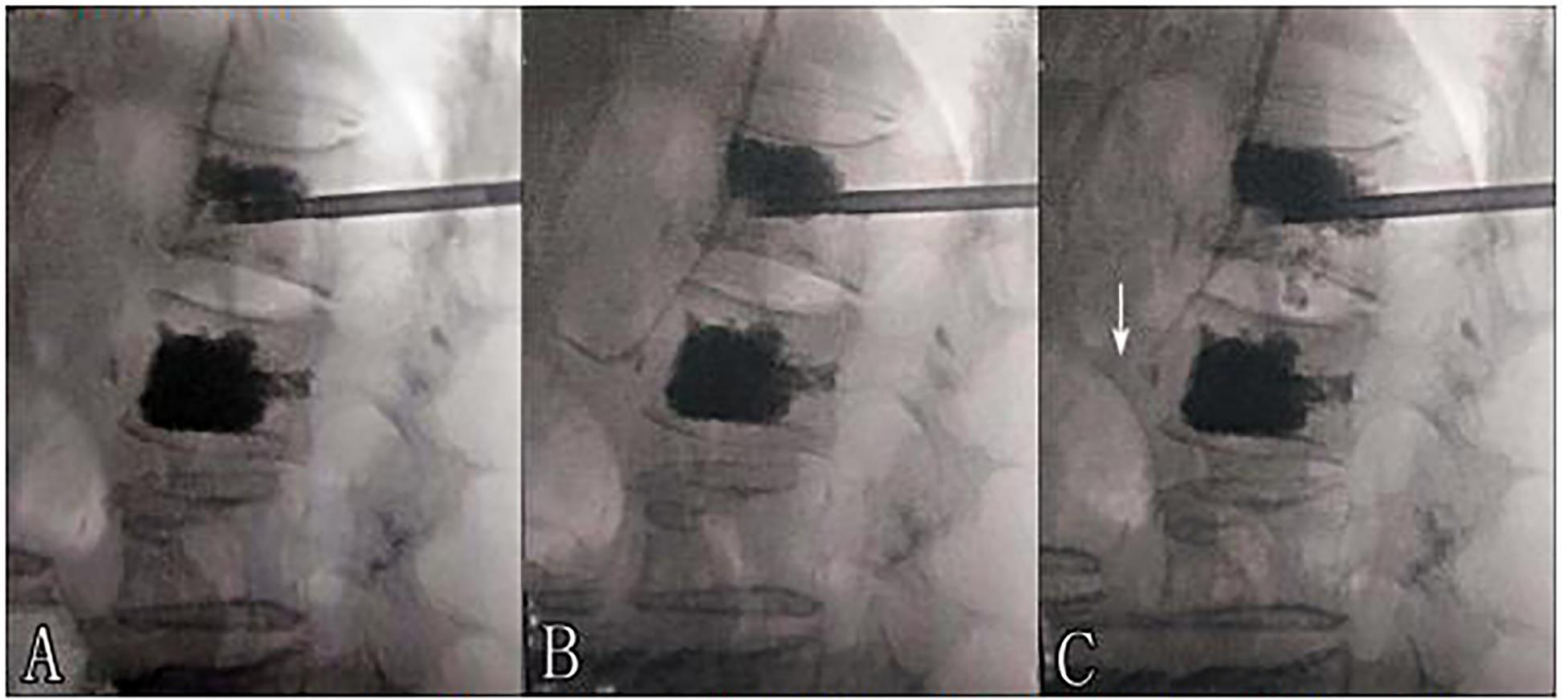
**(A,B)** Trajectory tracking of cement leakage into the paravertebral venous system was gradually prolonged with sustained injection. **(B,C)** Cement leakage migration. (Arrow: migration of the distal part of the prolonged cement leakage into the paravertebral venous system).

**Figure 2:**
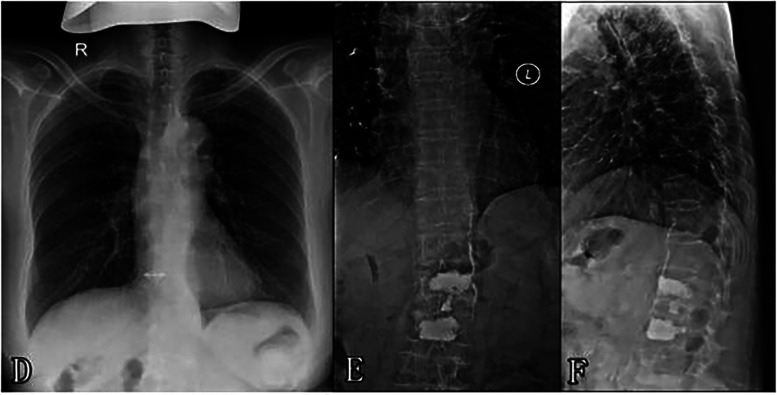
**(D)** Preoperative chest x-ray **(E,F)** postoperative thoracolumbar x-ray showing cement leakage into the paravertebral venous system and multiple cement emboli scattered throughout the lungs.

**Figure 3:**
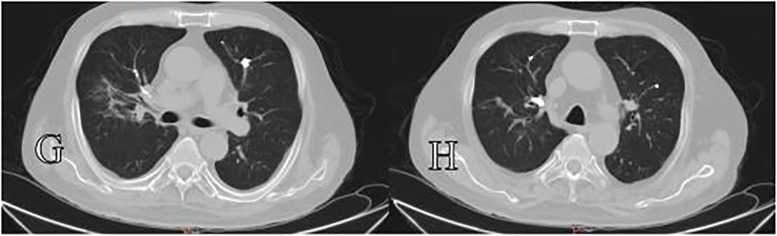
Panels **(G,H)** show chest computed tomography (CT) scans acquired more than one year postoperatively, which demostrated bone cement emboli within the pulmonary artery branches.

A 69-year-old woman was admitted to our hospital due to refractory back pain for approximately one month. Preoperative evaluation revealed that the patient had a bone mineral density T-score of −3.2 at L1-L4, indicating severe osteoporosis, imaging findings from MRI showing fresh osteoporotic vertebral compression fractures at L1 and L2 (characterized by T1-weighted hypointensity and T2-weighted hyperintensity with edema), while lower extremity Doppler ultrasound detected no pre-existing venous thrombosis. The routine preoperative medical evaluation was normal. Given her symptoms and spine images, she was treated with percutaneous vertebroplasty under local anesthesia (1% lidocaine) with continuous monitoring of heart rate, blood pressure, and oxygen saturation. Bone cement was typically infused into the vertebral body using a unilateral transpedicular approach, with intermittent anteroposterior and lateral fluoroscopy guidance. The percutaneous vertebroplasty procedure for L2 was completed successfully and approximately 4.5 ml of bone cement was infused (polymethylmethacrylate, viscosity: high, mixed for 3 min before injection). Then, the percutaneous vertebroplasty procedure for L1 was performed as follows. When the injection volume reached approximately 1.5 ml, anterior cement leakage in the paravertebral venous system was detected on lateral fluoroscopy. Immediately, vital signs were observed to be continuing smoothly with no obvious fluctuations (HR 72 bpm, BP 135/85 mmHg, SpO2 99%). Additionally, there were no complaints of cardiorespiratory discomfort by the patient. As the vertebral fill was inadequate, we continued the bone cement injection procedure. However, the trajectory tracking of cement leakage in the paravertebral venous system was gradually prolonged with continuous additional cement injection (Figure 1). When the cement injection dose reached approximately 4 ml, cement disk space extravasation was detected and we terminated the procedure at that time. The postoperative outcome was seemingly good and had no symptoms such as dyspnoea, coughing, haemoptysis, dizziness or palpitation. However, the postoperative thoracolumbar x-ray presented that multiple tubular and branching cement emboli were scattered throughout the lungs (Figure 2). Intraoperative lateral fluoroscopy data were analysed retrospectively, and we detected that sustained cement injection resulted in the migration of the distal part of the prolonged cement leakage tracks in the paravertebral venous system on the last lateral fluoroscopy. The subsequent treatment consisted of two days of conventional postoperative treatment and electrocardiogram monitoring, with no use of anticoagulation. The patient's vital signs continued smoothly until discharge. The patient was satisfied with the operation because her back pain was significantly relieved. With a one-year clinical follow-up after discharge, she remained asymptomatic. During the follow-up after discharge, it was noted that the patient developed pneumonia more than one year after leaving the hospital. Computed tomography (CT) at the local hospital confirmed the presence of bone cement emboli within the pulmonary artery branches, as illustrated in Figure 3, following successful treatment, the patient recovered uneventfully and was discharged from the hospital.

## Discussion

Percutaneous vertebroplasty has been widely used for the management of symptomatic vertebral compression fractures. The safety and effectiveness of percutaneous vertebroplasty have also been confirmed by several studies (15–17). However complications including cement leakage into the paravertebral venous system and even pulmonary cement embolism are sometimes reported. Although most patients with pulmonary cement embolism tend to be asymptomatic or only develop transient symptoms, there have been reports of serious and fatal outcomes. The migration of bone cement into the pulmonary arteries is considered to originate from the passage of cement into the paravertebral veins, followed by secondary migration into the azygos vein and, inferior vena cava, and ultimately into the pulmonary vasculature (6, 18). Several studies have identified that the presence of cement in the azygos vein or in the inferior vena cava are risk factors leading to pulmonary cement embolism (2, 19). An increased risk of cement embolization to the lungs with paravertebral venous cement leakage has also been found (6). Recent relevant studies have demonstrated that, for surgeries involving the thoracic vertebrae (T1–T9), a larger volume of bone cement injected into a single vertebra and the treatment of ≥3 vertebrae in a single operation are also risk factors for pulmonary bone cement embolism (20). In addition the risk of pulmonary embolism when bone cement leaks into the inferior vena cava is higher than when it leaks into the azygos vein (21). This case demonstrated that bone cement leakage into the paravertebral venous was associated with pulmonary cement embolism, which could provide direct clinical evidence. Because of the limited number of reports related to pulmonary cement embolism, there is no specified protocol for the treatment of pulmonary cement embolism to date. Antonio Krueger reviewed related literature and recommended clinical follow-up for asymptomatic patients or those with peripheral pulmonary cement embolism. The treatment included initial heparinization and a subsequent 6-month coumarin regimen for the treatment of thrombotic pulmonary embolisms was recommended for symptomatic or central embolisms. In addition, he deemed that surgical embolectomy should only be performed in exceptional cases with central embolisms and severe symptoms. Currently management based on the severity of symptoms and the size and location of the emboli is a wise choice. In clinical practice, attention should be paid to the following: the volume of bone cement injected into a single thoracic vertebra (T1–T9) should be controlled, with a recommended maximum of 3–4 ml, and high-viscosity cement is preferred to reduce leakage; for patients requiring treatment of ≥3 vertebrae, staged surgery (with an interval of ≥72 h) should be adopted to avoid cumulative risks of a single operation; intraoperative monitoring should be strengthened through multi-angle real-time fluoroscopy, and if bone cement leakage is detected, especially into paravertebral veins, the operation must be terminated immediately, so as to balance the surgical efficacy and safety (18, 22, 23). In this case, sustained injection resulted in clear cement leakage migration into the paravertebral veins, which likely led to the pulmonary cement embolism. Unfortunately, the risks of this type of misoperation do not attract enough attention. Although the patient was asymptomatic in the clinical follow-up, we should learn from the mistakes of this case.

## Conclusion

Cement leakage into the paravertebral venous system is associated with pulmonary cement embolism, and sustained injection is extremely dangerous when cement leakage into the paravertebral veins is detected during percutaneous vertebroplasty. In addition, we propose that once cement leakage into the paravertebral venous system is detected, percutaneous vertebroplasty should be terminated and that a secondary selective operation may be a better choice.

## Data Availability

The original contributions presented in the study are included in the article/Supplementary Material, further inquiries can be directed to the corresponding authors.

## References

[1] Galibert P Deramond H Rosat P Le Gars D. Preliminary note on the treatment of vertebral angioma by percutaneous acrylic vertebroplasty. Neurochirurgie. (1987) 33(2):166–8. 3600949

[2] Venmans A Klazen CA Lohle PN van Rooij WJ Verhaar HJ de Vries J Percutaneous vertebroplasty and pulmonary cement embolism: results from VERTOS II. AJNR Am J Neuroradiol. (2010) 31(8):1451–3. 10.3174/ajnr.A2127 20488908 PMC7966118

[3] Wang LJ Yang HL Shi YX Jiang WM Chen L. Pulmonary cement embolism associated with percutaneous vertebroplasty or kyphoplasty: a systematic review. Orthop Surg. (2012) 4(3):182–9. 10.1111/j.1757-7861.2012.00193.x 22927153 PMC6583132

[4] Mansour A Abdel-Razeq N Abuali H Makoseh M Shaikh-Salem N Abushalha K Cement pulmonary embolism as a complication of percutaneous vertebroplasty in cancer patients. Cancer Imaging. (2018) 18(1):5. 10.1186/s40644-018-0138-8 29422089 PMC5806228

[5] Leitman D Yu V Cox C. Investigation of polymethylmethacrylate pulmonary embolus in a patient ten years following vertebroplasty. J Radiol Case Rep. (2011) 5(10):14–21. 10.3941/jrcr.v5i10.815 22470765 PMC3303462

[6] Habib N Maniatis T Ahmed S Kilkenny T Alkaied H Elsayegh D Cement pulmonary embolism after percutaneous vertebroplasty and kyphoplasty: an overview. Heart Lung. (2012) 41(5):509–11. 10.1016/j.hrtlng.2012.02.008 22425258

[7] Geraci G Lo Iacono G Lo Nigro C Cannizzaro F Cajozzo M Modica G. Asymptomatic bone cement pulmonary embolism after vertebroplasty: case report and literature review. Case Rep Surg. (2013) 2013:591432. 10.1155/2013/591432 23738182 PMC3662203

[8] Huh S Lee H. Pulmonary bone cement embolism: CT angiographic evaluation with material decomposition using gemstone spectral imaging. Korean J Radiol. (2014) 15(4):443–7. 10.3348/kjr.2014.15.4.443 25053903 PMC4105806

[9] Makary MS Zucker IL Sturgeon JM. Venous extravasation and polymethylmethacrylate pulmonary embolism following fluoroscopy-guided percutaneous vertebroplasty. Acta Radiol Open. (2015) 4(8):2058460115595660. 10.1177/2058460115595660 26331092 PMC4548728

[10] Chang CY Huang SF. Asymptomatic pulmonary cement embolism. CMAJ. (2017) 189(14):E543. 10.1503/cmaj.160579 28396332 PMC5386849

[11] Lamparello NA Jaswani V DeSousa K Shapiro M Kovacs S. Percutaneous retrieval of an embolized kyphoplasty cement fragment from the pulmonary artery: a case report and literature review. J Radiol Case Rep. (2016) 10(7):40–7. 10.3941/jrcr.v10i7.2806 27761188 PMC5065278

[12] Yuan Z Zhou Y Zhou X Liao X. Severe pulmonary embolism was secondary to cement inferior vena cava embolism after percutaneous vertebroplasty. Ann Vasc Surg. (2018) 48:255.e1–e3. 10.1016/j.avsg.2018.01.003 29428532

[13] D'Errico S Niballi S Bonuccelli D. Fatal cardiac perforation and pulmonary embolism of leaked cement after percutaneous vertebroplasty. J Forensic Leg Med. (2019) 63:48–51. 10.1016/j.jflm.2019.03.004 30861473

[14] Drigalla D Stone CK Juergens AL. Delayed symptomatic pulmonary embolism secondary to bone cement after percutaneous vertebroplasty. J Emerg Med. (2021) 60(3):e45–7. 10.1016/j.jemermed.2020.10.045 33419654

[15] Lamy O Uebelhart B Aubry-Rozier B. Risks and benefits of percutaneous vertebroplasty or kyphoplasty in the management of osteoporotic vertebral fractures. Osteoporos Int. (2014) 25(3):807–19. 10.1007/s00198-013-2574-4 24264371

[16] Xie L Zhao ZG Zhang SJ Hu YB. Percutaneous vertebroplasty versus conservative treatment for osteoporotic vertebral compression fractures: an updated meta-analysis of prospective randomized controlled trials. Int J Surg. (2017) 47:25–32. 10.1016/j.ijsu.2017.09.021 28939236

[17] Zuo XH Zhu XP Bao HG Xu CJ Chen H Gao XZ Network meta-analysis of percutaneous vertebroplasty, percutaneous kyphoplasty, nerve block, and conservative treatment for nonsurgery options of acute/subacute and chronic osteoporotic vertebral compression fractures (OVCFs) in short-term and long-term effects. Medicine (Baltimore). (2018) 97(29):e11544. 10.1097/MD.0000000000011544 30024546 PMC6086478

[18] Lee IJ Choi AL Yie MY Yoon JY Jeon EY Koh SH CT evaluation of local leakage of bone cement after percutaneous kyphoplasty and vertebroplasty. Acta Radiol. (2010) 51(6):649–54. 10.3109/02841851003620366 20528649

[19] Kim YJ Lee JW Park KW Yeom JS Jeong HS Park JM Pulmonary cement embolism after percutaneous vertebroplasty in osteoporotic vertebral compression fractures: incidence, characteristics, and risk factors. Radiology. (2009) 251(1):250–9. 10.1148/radiol.2511080854 19332856

[20] Sun HB Jing XS Shan JL Bao L Wang DC Tang H. Risk factors for pulmonary cement embolism associated with percutaneous vertebral augmentation: a systematic review and meta-analysis. Int J Surg. (2022) 101:106632. 10.1016/j.ijsu.2022.106632 35452848

[21] Sun X Deng M Xu W Yang H Liu A Meng X CT features and risk factors of pulmonary cement embolism after vertebroplasty or kyphoplasty in patients with vertebral compression fracture: a retrospective cohort study. Quant Imaging Med Surg. (2023) 13(4):2397–407. 10.21037/qims-22-569 37064367 PMC10102772

[22] Krueger A Bliemel C Zettl R Ruchholtz S. Management of pulmonary cement embolism after percutaneous vertebroplasty and kyphoplasty: a systematic review of the literature. Eur Spine J. (2009) 18(9):1257–65. 10.1007/s00586-009-1073-y 19575243 PMC2899525

[23] Zhao Y Liu T Zheng Y Wang L Hao D. Successful percutaneous retrieval of a large pulmonary cement embolus caused by cement leakage during percutaneous vertebroplasty: case report and literature review. Spine (Phila Pa 1976). (2014) 39(26):E1616–21. 10.1097/BRS.0000000000000613 25271513

